# Laparoscopic Adjustable Gastric Banding—Should a Second Chance Be Given?

**DOI:** 10.1007/s11695-020-04613-1

**Published:** 2020-04-18

**Authors:** Bianca M. Leca, Uzma Khan, Jenny Abraham, Louise Halder, Emma Shuttlewood, Neha Shah, Hugh L. Ellis, Simon J. B. Aylwin, Thomas M. Barber, Vinod Menon, Harpal S. Randeva, Georgios K. Dimitriadis

**Affiliations:** 1 Department of Endocrinology, Diabetes and Metabolism - WISDEM Centre, University Hospitals Coventry and Warwickshire NHS Trust, Coventry, CV2 2DX UK; 2 Department of Surgery, University Hospitals Coventry and Warwickshire NHS Trust, Coventry, CV2 2DX UK; 3 Department of Dietetics, University Hospitals Coventry and Warwickshire NHS Trust, Coventry, CV2 2DX UK; 4 Department of Endocrinology and Metabolic Medicine, King’s College Hospital NHS Foundation Trust, Denmark Hill, London, SE5 9RS UK; 5 Clinical Sciences Research Laboratories, University of Warwick Medical School, Coventry, CV2 2DX UK

**Keywords:** Laparoscopic adjustable gastric banding, Bariatric surgery, Obesity, T2DM, Hyperlipidemia, Hypertension, Weight loss

## Abstract

**Background:**

Obesity is a chronic relapsing-remitting disease and a global pandemic, being associated with multiple comorbidities. Laparoscopic adjustable gastric banding (LAGB) is one of the safest surgical procedures used for the treatment of obesity, and even though its popularity has been decreasing over time, it still remains an option for a certain group of patients, producing considerable weight loss and improvement in obesity-associated comorbidities.

**Methods:**

The aim of this study was to evaluate the impact of weight loss following LAGB on obesity-associated comorbidities, and to identify factors that could predict better response to surgery, and patient sub-groups exhibiting greatest benefit. A total of 99 severely obese patients (81.2% women, mean age 44.19 ± 10.94 years, mean body mass index (BMI) 51.84 ± 8.77 kg/m^2^) underwent LAGB in a single institution. Results obtained 1, 2, and 5 years postoperatively were compared with the pre-operative values using SPPS software version 20.

**Results:**

A significant drop in BMI was recorded throughout the follow-up period, as well as in A1c and triglycerides, with greatest improvement seen 2 years after surgery (51.8 ± 8.7 kg/m^2^ vs 42.3 ± 9.2 kg/m^2^, *p* < 0.05, 55.5 ± 19.1 mmol/mol vs 45.8 ± 13.7 mmol/mol, *p* < 0.05, and 2.2 ± 1.7 mmol/l vs 1.5 ± 0.6 mmol/l). Better outcomes were seen in younger patients, with lower duration of diabetes before surgery, and lower pre-operative systolic blood pressure.

**Conclusions:**

Younger age, lower degree of obesity, and lower severity of comorbidities at the time of surgery can be important predictors of successful weight loss, making this group of patients the ideal candidates for LAGB.

## Introduction

Laparoscopic adjustable gastric banding (LAGB) includes the placement of a silicone ring around the stomach to create a small upper gastric pouch at the bottom of the esophagus. This procedure was introduced in the 1970s and remains safe, well tolerated, and efficacious with a relative low risk of serious complications. Another benefit to this procedure is the ability to adjust the band enhancing its weight loss effect without compromising safety. Furthermore, LAGB is a reversible form of laparoscopic surgery making it an attractive option for the majority of patients, although it is not promoted as a temporary procedure due to the significant risk of weight regain after removal [1, 2].

LAGB is ideally placed on the cardia of the stomach, just below the esophagogastric junction. It is assumed that the presence of a band in this position causes a meal to accumulate in the pouch of stomach proximal to it, before gradually being released into the remainder of the gut. Thus, the band is thought to work by restricting the volume of food ingested to that able to be accommodated in the proximal pouch. This small volume of food was thought to stretch the stomach and cause early satiety. Gradual emptying of the proximal pouch into the infra-band stomach is thought to be responsible for prolonged inter-meal satiety [3]. More recently, it has been hypothesized that LAGB mechanism of action includes the induction of early and prolonged satiety; however, the intraluminal events that lead to this are far more complex than simple retention of food in the proximal pouch and merit further evaluation beyond the scope of this manuscript [4].

Despite the above, clinical practice is moving away from LAGB due to lower efficacy and high conversion to other forms of surgery. Patients with dysphagia and/or regurgitation, or poor weight loss response in the context of LAGB should be evaluated for pseudoachalasia [5].

LAGB, nevertheless, remains a safe and effective treatment, and it is crucial to identify specific patient characteristics that predispose to good metabolic response to LAGB as this can be invaluable in the optimization of current and development of future tailored metabolic interventions to treat obesity.

## Materials and Methods

A retrospective analysis of 99 patients who underwent LAGB over a period of 3 years was conducted. The study received favorable ethical approval (GafREC ref: GF0366) by local authorities.

Eligible for surgery were patients who met the National Institute for Health and Care Excellence (NICE) clinical guideline CG 189, with a body mass index (BMI) of ≥ 35 kg/m^2^ and established obesity-related comorbidities including type 2 diabetes mellitus (T2DM), obstructive sleep apnea (OSA), hyperlipidemia, or other that could be improved following weight loss, or ≥ 40 kg/m^2^ without any comorbidities, unable to achieve or maintain adequate weight loss following appropriate non-surgical interventions.

A standard LAGB procedure using pars flaccida technique was performed in all cases by the same two experienced bariatric surgeons. All patients were discharged on the first day following surgery and band adjustments were performed at scheduled intervals as per NICE guidance.

Clinical and biochemical assessments were performed at baseline, 1, 2, and 5 years postoperatively. Weight and height were measured in light clothing, and without shoes. Weight loss after surgery was calculated using both total weight loss (%TWL), and excess weight loss (%EWL). %TWL was obtained applying the formula: (initial weight – post-op weight) × 100/initial weight. %EWL was calculated by dividing the number of kilograms lost by pre-operative excess body weight (EBW), assuming a healthy BMI at 25 kg/m^2^. Blood pressure was measured using Welch Allyn 7000-APM blood pressure meter. The measurement was done twice, with the patient seated for at least 10 min, and the average was considered for analysis. Blood samples were obtained after at least 8-h overnight fast to evaluate glucose and lipid metabolism. The change in different blood parameters (Δ) was expressed in percentages out of the pre-operative values, following the formula: (Δ parameter) = (parameter value baseline − parameter value postoperatively) × 100/parameter value pre-op.

The presence of T2DM, hypertension, dyslipidemia, and OSA was assessed based on patients’ medical records, clinical examination, and blood test results. The improvement in obesity-associated comorbidities was evaluated 1, 2, and 5 years postoperatively. The use of glucose lowering agents, antihypertensive medication, and lipid lowering drugs was recorded at each hospital visit.

Statistical analysis was performed using Statistical Package for the Social Sciences Software (SPSS) version 20. Data was reported as mean ± SD for continuous variables and in percentages for categorial variables. The comparisons between parameters were carried out using parametric (paired sample *T* test, independent sample *T* test) or non-parametric tests (Chi-squared test), and correlations were performed using Pearson analysis. A *p* value < 0.05 was considered statistically significant.

## Results

A total of 99 morbidly obese patients (17 men and 82 women) underwent LAGB over a 3-year period. The patients had a mean age at the time of surgery of 44.19 ± 10.94 years (range 22–71 years), and a mean BMI of 51.84 ± 8.77 kg/m^2^ (54.89 ± 14.21 kg/m^2^ in men versus 51.22 ± 7.17 kg/m^2^ in women, *p* = 0.013). Patients had a mean EBW of 74.74 ± 13.38 kg, ranging between 25 and 122.8 kg. The prevalence of comorbidities was 42.4% for T2DM, 57.4% for hypertension, 28.7% for dyslipidemia, and 26.3% were diagnosed with OSA.

Thirty-four of the patients included in the study had their band removed at 5.5 ± 2.25 years after insertion, due to complications. The main complication recorded was ineffective weight loss, followed by slippage, erosion, and infection. Data obtained after band removal was not included in our analysis. There were no deaths attributable to the procedure. Baseline, 1, 2, and 5 years data are presented in Table 1.

**Table 1:** Sample general characteristics

Parameter	Baseline	1 year post-op	*P* value	2 years post-op	*P* value	5 years post-op	*P* value
BMI (kg/m^2^)	51.84 ± 8.77	43.99 ± 8.02	< 0.001	42.35 ± 9.26	< 0.001	43.40 ± 8.34	< 0.001
SBP (mmHg)	144.75 ± 17.63	143.32 ± 20.03	ns	140.58 ± 20.98	ns	140.55 ± 21.60	ns
DBP (mmHg)	78.61 ± 11.86	76.70 ± 11.16	ns	78.55 ± 13.37	ns	77.53 ± 10.92	ns
A1c (mmol/mol)	55.54 ± 19.11	45.71 ± 14.38	0.001	45.81 ± 13.73	0.002	49.75 ± 17.70	ns
Total Cho (mmol/l)	4.79 ± 0.96	4.94 ± 1.04	ns	4.93 ± 1.01	ns	4.94 ± 0.97	ns
HDL Cho (mmol/l)	1.33 ± 0.41	1.45 ± 0.42	0.037	1.52 ± 0.44	< 0.001	1.52 ± 0.45	0.003
LDL Cho (mmol/l)	2.53 ± 0.83	2.71 ± 0.99	ns	2.68 ± 0.98	ns	2.56 ± 0.97	ns
TG (mmol/l)	2.21 ± 1.76	1.69 ± 1.07	0.039	1.50 ± 0.66	0.006	1.85 ± 1.21	ns

*BMI* body mass index, *SBP* systolic blood pressure, *DBP* diastolic blood pressure, HDL high-density lipoprotein, LDL low-density lipoprotein, TG triglycerides

A decrease in medication use was recorded throughout the follow-up period, and results are presented in Table 2. Statistically significant was the decrease in the use of antihyperglycemic and antihypertensive medication at 1 and 2 years postoperatively.

**Table 2:** Medication use

Medication use	Baseline	1 year post-op	*P* value	2 years post-op	*P* value	5 years post-op	*P* value
Glucose-lowering agents	36.8%	26.1%	0.025	27%	0.046	23.2%	ns
Antihypertensive drugs	57.4%	53.8%	0.018	54.8%	0.004	52.9%	ns
Lipid-lowering agents	28.7%	25.4%	ns	24.3%	ns	22.1%	ns

**Fig. 1:**
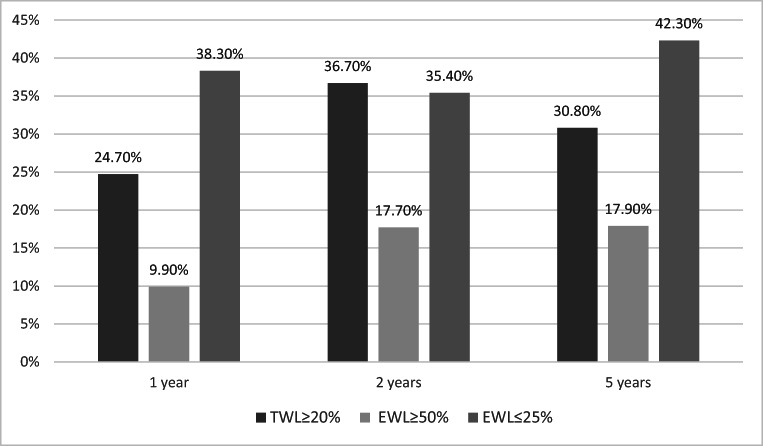
The effectiveness/ineffectiveness of the procedure was assessed using %EWL (≥ 50% or ≤ 25%) and %TWL (≥ 20%)

In regard to weight loss, results of mean %EWL calculated at 1, 2, and 5 years postoperatively were 29.91 ± 17.06%, 36.96 ± 25.65%, and 29.97 ± 25.68%, respectively, while mean %TWL was 15.3 ± 8.51%, 18.57 ± 13.19%, and 15.63 ± 13.57%. The effectiveness of the procedure was assessed based either on %EWL (≥ 50%, or ≤ 25%), or %TWL (≥ 20%) (Fig. 1).

**Fig. 2:**
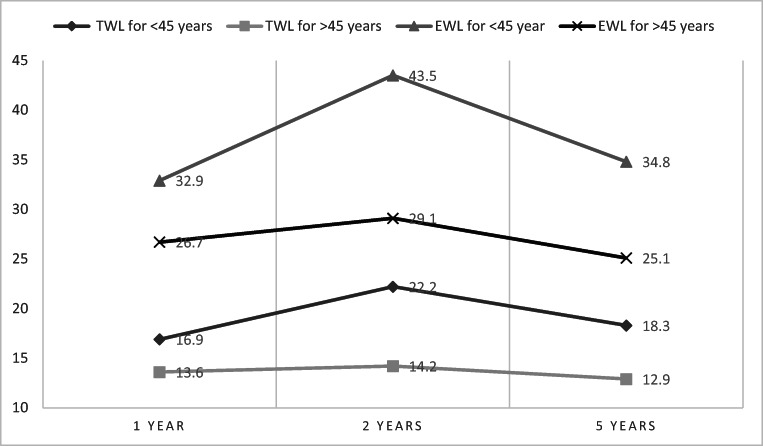
Younger patients lost more weight through the follow-up period, with statistically significant results obtained 2 years after surgery

In subgroup analysis, using gender or baseline BMI as possible predictors of a good response to surgery, no statistically significant differences in the amount of %TWL or %EWL were found. In contrast, younger patients lost more weight through the follow-up period, with statistically significant results obtained 2 years after surgery (Fig. 2). The data is presented in Tables 3 and 4.

**Table 3:** %EWL in subgroup analysis

	1 year	*P* value	2 years	*P* value	5 years	*P* value
% EWL by gender						
Men	33.83 ± 9.02	ns	36.47 ± 11.73	ns	27.00 ± 13.17	ns
Women	29.16 ± 18.15		37.06 ± 27.64		30.62 ± 27.71	
% EWL by age						
< 45 years	32.96 ± 20.10	ns	43.58 ± 29.98	0.011	34.81 ± 29.05	ns
≥ 45 years	26.78 ± 12.77		29.05 ± 16.42		25.13 ± 21.08	
% EWL by initial BMI						
< 50 kg/m^2^	31.22 ± 21.46	ns	35.82 ± 25.78	ns	27.48 ± 29.92	ns
≥ 50 kg/m^2^	29.09 ± 13.84		37.74 ± 25.81		31.28 ± 23.34	

*EWL* excess weight loss, *BMI* body mass index

**Table 4:** %TWL in subgroup analysis

	1 year	*P* value	2 years	*P* value	5 years	*P* value
% TWL by gender						
Men	17.37 ± 4.44	ns	18.62 ± 6.23	ns	14.28 ± 8.36	ns
Women	14.9 ± 9.05		18.56 ± 14.2		15.93 ± 14.5	
% TWL by age						
< 45 years	16.9 ± 9.81	ns	22.22 ± 15.6	0.006	18.31 ± 15.40	ns
≥ 45 years	13.65 ± 6.67		14.21 ± 7.74		12.95 ± 11.02	
% TWL by initial BMI						
< 50 kg/m^2^	14.34 ± 9.85	ns	15.72 ± 11.61	ns	12.03 ± 12.49	ns
≥ 50 kg/m^2^	15.9 ± 7.61		20.5 ± 13.95		17.54 ± 13.85	

*TWL* total weight loss, *BMI* body mass index

Further exploration of the data available established a negative correlation between the duration of T2DM at surgery and both 1 year %TWL (*r* = − 0.362, *p* = 0.028) and %EWL (*r* = − 0.393, *p* = 0.016), with poorer outcomes in patients with longer duration of T2DM before surgery. Another determinant of %EWL was the value of pre-operative systolic blood pressure, a weak negative correlation being established between 2 years %EWL and pre-op systolic blood pressure (SBP) (*r* = − 0.247, *p* = 0.036). Two years after surgery, %EWL was higher in patients without diabetes or hypertension, but not statistically significant. Results are shown in Table 5.

**Table 5:** %EWL in the presence of obesity-associated comorbidities

	2 years % EWL	*P* value
Presence of T2DM		
No	39.17 ± 20.75	ns
Yes	34.32 ± 17.86	
Presence of hypertension		
No	43.87 ± 23.33	ns
Yes	33.14 ± 19.73	

*T2DM* type 2 diabetes mellitus, *EWL* excess weight loss

When analyzing the improvement in metabolic profile 2 and 5 years postoperatively, better outcomes were noted in patients with a lower BMI at baseline. Negative correlations were established between initial BMI and % A1c and % triglycerides (TG) at 2 and 5 years after surgery. Results are presented in Table 6.

**Table 6:** Correlations between baseline BMI and change in A1c and triglycerides

	*r*	*p*
2 years %A1c	− 0.341	0.023
5 years %A1c	− 0.484	0.001
2 years %TG	− 0.591	0.001
5 years %TG	− 0.440	0.006

*A1c* glycosylated hemoglobin, *TG* triglycerides, *BMI* body mass index

## Discussion

With the continuous rise in the prevalence of obesity worldwide, choosing the ideal treatment is both a challenge and a necessity. All bariatric surgery procedures lead to substantial weight loss and improvement in obesity-associated comorbidities, although the rate of complications and long-term results can be highly variable. The key to obtaining the best outcomes is a personalized approach, which consists of identifying the patients who could benefit the most from the currently available surgical options.

Even though the popularity of LAGB has been declining rapidly in the past years due to high rates of ineffective weight loss and frequent re-operations [3], the technical ease of the procedure, the low early postoperative complications, and the short length of hospitalization [3] could make it a feasible option in a selected group of patients.

In the studied population, the mean initial BMI of 51.84 kg/m^2^ was significantly higher compared with existing data. O’Brien et al. report a mean baseline BMI of 43.2 kg/m^2^ when analyzing a total of 8378 patients who underwent LAGB [4]. In addition, the prevalence of obesity-related comorbidities was slightly higher than in other studies [6]. In a meta-analysis which included 161,756 patients who underwent bariatric surgery, the prevalence of T2DM was 26%, hypertension 47%, dyslipidemia 28%, and 25% for sleep apnea [6], compared with our findings of 42.4%, 57.4%, 28.7%, and 26.3% respectively.

In accordance with the existing literature [4], no deaths attributable to the procedure were recorded, confirming the safety of LAGB, which has the lowest mortality and complications rates compared with the other surgical interventions. However, in contrast to other studies, higher reoperation rates were found as the percentage of patients who had the band removed due to complications was 34.34%, where the explantation rates were 8.6% in a 20-year follow-up period of 3554 patients [4], 3.7% in 1791 obese patients followed up for 12 years [7]. Similar removal rates of 34.2% at 10 years and 46.7% at 15 years were reported by Carandina et al. when analyzing data from 301 patients who underwent LAGB [8]. Arapis et al., in a study which included 897 patients, reported a band failure of more than 70% at 15 years [9].

A statistically significant decrease in BMI from 51.82 to 43.99 kg/m^2^, 42.35 kg/m^2^, and 43.40 kg/m^2^ was recorded 1, 2, and 5 years postoperatively, with patients remaining morbidly obese. In regard to the metabolic parameters, A1c dropped, but with a mean value after surgery still in the prediabetic/diabetic range (from 55.54 to 45.71 mmol/mol, 45.81 mmol/mol, 49.75 mmol/mol), and high-density lipoprotein (HDL) cholesterol slightly increased thought the follow-up period. No change in total and low-density lipoprotein (LDL) cholesterol was noted, and TG improved 2 years after surgery when weight loss was maximal. These findings suggest that the amount of weight loss was not sufficient to reach healthy BMI range, nor to achieve significant improvement in metabolic parameters. Others report better outcomes after 36 months in a study of 290 patients, with a significant decrease in blood pressure, glucose, and cholesterol levels [10]. However, the study population was younger (20–55 years old), had lower initial BMI levels, and lower rates of comorbidities [10]. Similar findings were reported by Steffen et al. in a 7-year prospective study on 388 patients undergoing LAGB [11]. In a metanalysis by Li et al., rates of hypertension improvement were similar to those seen after laparoscopic sleeve gastrectomy (61% versus 63%) [12]. In contrast, Aarts et al., after analyzing data from 201 patients followed up for 14 years, noticed that comorbidities returned or patients developed new ones, despite initially observed improvement [13].

Mean %EWL at 1, 2, and 5 years postoperatively was 29.91%, 36.96%, and 29.97%, which is lower than in the existing literature. In a recent paper on 3566 patients by Furbetta et al., a mean of 49%, 53.6%, and 59.2% EWL was recorded at 10, 15, and 20 years [14], demonstrating that LAGB can be a highly effective surgical treatment of obesity when a multidisciplinary approach is used. In the existing studies, EWL% ranged between 27 and 65.7%, with a follow-up duration from 10 to 16 years [4]. Mean %TWL of 15.3%, 18.57%, and 15.63% at 1, 2, and 5 years after surgery was comparable with results obtained by O’Brien et al. [4] at the same time points: 18.1%, 20.4%, and 19.5%. Maximum weight loss was reached 2 years after surgery, using any of %EWL or %TWL, result comparable with other studies [4].Chang et al. [6] reported in their meta-analysis that younger age and pre-surgery BMI are positively correlated with the amount of weight loss. Similar results were noted in our current study. Patients aged < 45 years lost more weight, results which were maintained through the follow-up period. Initial BMI had an impact on improvement of comorbidities, not on the amount of weight loss. Patients with lower pre-operative weight had a better improvement in glucose and lipid profile at 2 and 5 years after surgery, suggesting that baseline BMI should be an important factor when choosing LAGB as an obesity treatment. A systematic review looking at the outcomes of LAGB in patients with BMI ≤ 35 kg/m^2^ reported up to 71.9% EWL at 5 years, with partial or total resolution of comorbidities [15]. Other studies reported better results in male and older patients [16] or in those with higher initial BMI [14, 16].

Limitations of this study include the retrospective design, relatively small sample size, lack of quantifiable information on the type and level of support these patients received from the local bariatric multidisciplinary team, and lack of any measures of psychological wellbeing which may have impacted on weight loss postoperatively.

In the presence of obesity-associated comorbidities, like diabetes or hypertension, gastric band surgery proved to be less effective, also confirmed by other studies [17]. Additionally, longer duration of diabetes at the time of surgery and higher systolic blood pressure were negatively correlated with the amount of weight loss. These findings indicate that LAGB is more beneficial in patients without comorbidities at the time of surgery, in line with the procedure’s restrictive nature.

## Conclusions

In order to improve the care of patients with obesity, careful consideration should be given before choosing between the available surgical procedures. LAGB has proven to be a safe, reversible, and less invasive technique that can lead to effective weight loss and improvement of comorbidities. This study found that younger age, lower degree of obesity, and lower severity of comorbidities at the time of surgery may be important predictors of successful weight loss, making this group of patients the ideal candidates for LAGB.
